# Case Report: Diagnostic overlap of OHVIRA syndrome and Gartner duct cyst: challenges in imaging and management

**DOI:** 10.3389/fped.2025.1536314

**Published:** 2025-05-22

**Authors:** Junfeng Zhao, Yaqing Bao, Linglu Gong, Zhan Shi, Xiaoqiang Chen, Yan Li

**Affiliations:** ^1^ Department of Pediatric Urology, Ningbo University Affiliated Women and Children’s Hospital, Ningbo, China; ^2^ Department of Pharmacy, Ningbo University Affiliated Women and Children’s Hospital, Ningbo, China; ^3^ Department of Ultrasound Medicine, Ningbo University Affiliated Women and Children’s Hospital, Ningbo, China

**Keywords:** OHVIRA, Gartner duct cyst/GDC, ectopic ureter, oblique septum, renal agenesis

## Abstract

This case series explores the diagnostic overlap between OHVIRA syndrome (obstructed hemivagina and ipsilateral renal anomaly) and Gartner duct cyst (GDC) with ipsilateral renal dysplasia in two prepubertal girls. Both cases exhibited similar imaging features on CT and MRI—including unilateral renal agenesis, ectopic ureter with abnormal insertion, cystic lesions posterolateral to the bladder— which led to significant diagnostic challenges. Despite differing in pathology, the overlap in embryologic origins and imaging findings made differentiation difficult. This series underscores the importance of comprehensive imaging and a multidisciplinary approach for accurate diagnosis. Current management strategies are discussed, highlighting the need for individualized treatment plans in prepubertal patients with complex genitourinary anomalies.

## Introduction

OHVIRA syndrome (Obstructed Hemivagina and Ipsilateral Renal Anomaly) and Gartner duct cyst (GDC) are rare congenital anomalies of the genitourinary system, both arising from abnormalities in the Müllerian and/or Wolffian ducts (1–3). OHVIRA syndrome typically presents with an obstructed hemivagina, uterine anomalies, and ipsilateral renal agenesis. GDCs are remnants of the Wolffian duct that develop in the anterolateral vaginal wall and may extend toward the posterior aspect of the bladder (4). Notably, approximately 10% of GDCs are associated with renal agenesis or dysplasia, which is often accompanied by an ectopic ureter (4, 5). Such ectopic ureters are also commonly observed in OHVIRA syndrome (3). Therefore, when both conditions are associated with an ectopic ureter, overlapping imaging features may lead to significant diagnostic challenges.

We describe two prepubertal girls who were referred to our tertiary hospital for evaluation and management, presented one month apart (Supplementary Table S1). These cases highlight the diagnostic complexities in distinguishing these two anomalies and underscore the importance of imaging analysis supported by a multidisciplinary approach for appropriate management. Written informed consent was obtained from the patients and their families for publication of these cases.

## Case descriptions

### Case 1: OHVIRA syndrome with ectopic ureter

**Figure 1:**
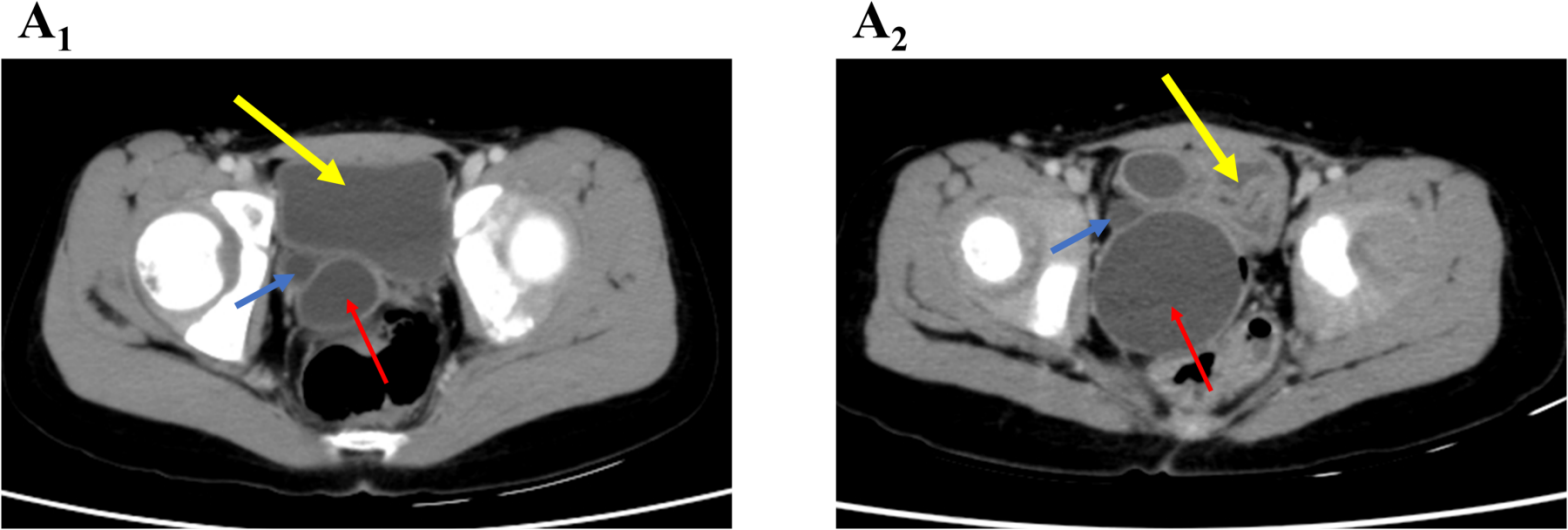
Axial CT image showing a cystic mass (red arrow) and a dilated ureter (blue arrow), both located posterior and lateral to the bladder (yellow arrow) on the right side. **(A_1_)** OHVIRA syndrome case; **(A_2_)** Gartner duct cyst case.

An 8-year-old girl was referred for evaluation after prenatal ultrasound revealed right renal ectopia with multicystic dysplasia. Her growth and development were normal. One year ago, a contrast-enhanced abdominal computed tomography (CT) revealed right renal dysplasia, with compensatory hypertrophy of the left kidney (Supplementary Figure S1A). Dilation of the distal ureter behind the bladder and a cystic mass located between the bladder and rectum suggested vaginal fluid accumulation (Figure 1A_1_, Supplementary Figure S1B). Due to the absence of symptoms, outpatient follow-up was recommended.

**Figure 2:**
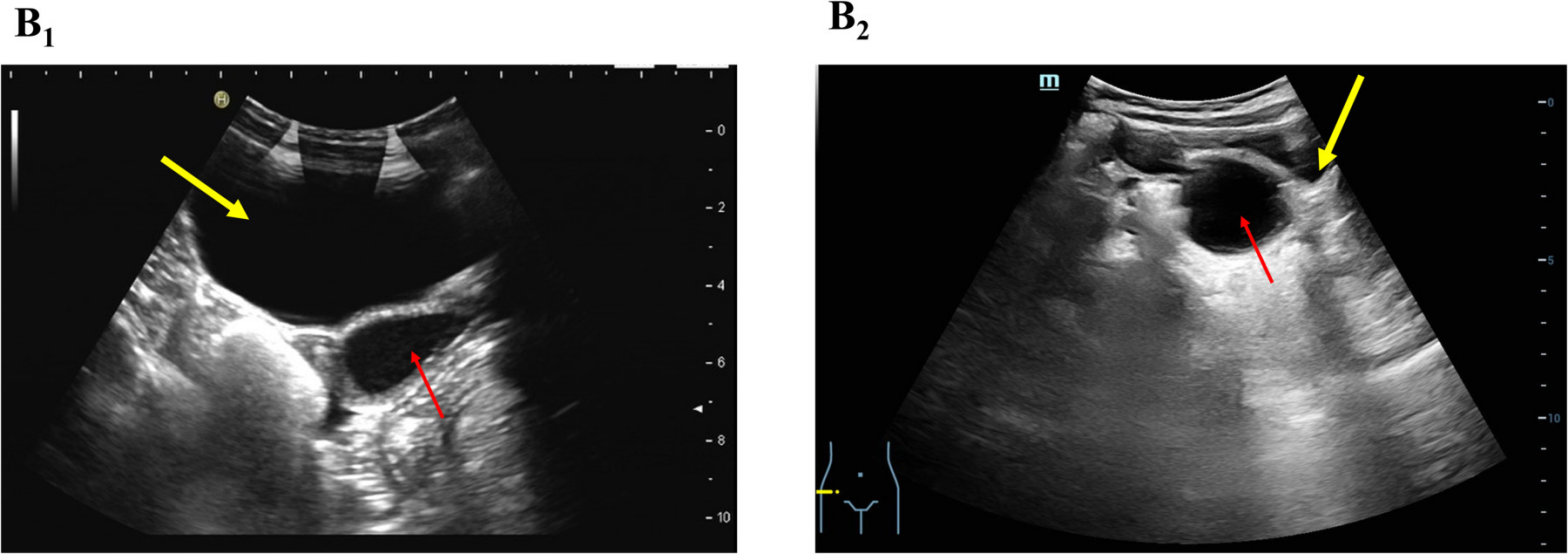
B-ultrasound showing a cystic mass (red arrow) posterior to the bladder (yellow arrow), likely originating from the vagina. **(B_1_)** OHVIRA syndrome case; **(B_2_)** Gartner duct cyst case.

**Figure 3:**
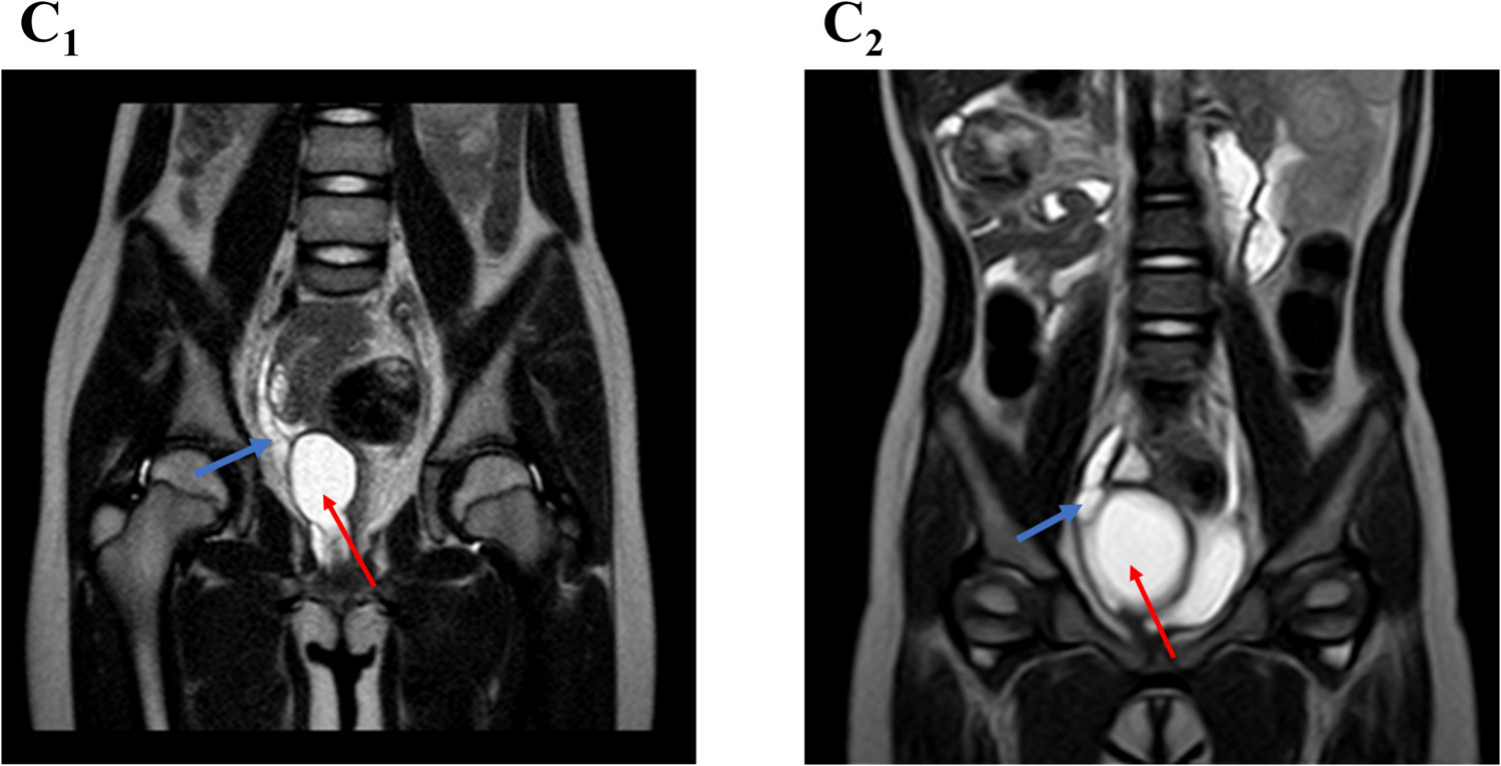
Sagittal MRI image showing a dilated right distal ureter (blue arrow) adjacent to a cyst (red arrow), possibly ectopically opening into the cyst. **(C_1_)** OHVIRA syndrome case; **(C_2_)** Gartner duct cyst case.

Upon admission, physical examination was unremarkable. Ultrasound revealed a uterus measuring 21 × 10 × 19 mm and a cystic mass (33 × 24 mm) posterior to the bladder, closely related to the vagina (Figure 2B_1_). The distal right ureter (9 mm) appeared to communicate with the vagina (Supplementary Figure S2). Magnetic resonance imaging (MRI) indicated a cystic lesion posterior to the bladder, likely due to vaginal distension from an oblique septum (Supplementary Figures S3A,B). The dilated right distal ureter appeared to ectopically insert into the vagina (Figure 3C_1_).

A multidisciplinary team, including radiologists, gynecologists, and pediatric urologists, consulted on the case. The surgical team performed cystoscopy, vaginoscopic septum resection, and laparoscopic removal of the dysplastic kidney and ureter. Cystoscopy revealed the absence of the right ureteral orifice, while the left appeared normal. Vaginoscopy identified and resected an oblique septum compressing the upper right vaginal wall, exposing a hemivagina merging distally with the original vagina. Laparoscopy successfully removed the ectopic dysplastic kidney and distal ureter. The patient recovered uneventfully and was discharged on postoperative day three. The patient and her family were satisfied with the treatment outcome, noting a smooth recovery and effective care. A three-month follow-up ultrasound showed no vaginal fluid accumulation, and the patient remained asymptomatic.

### Case 2: Gartner duct cyst with ectopic ureter and renal dysplasia

A 2-year-7-month-old girl was referred for evaluation following prenatal ultrasound findings of right renal ectopia with dysplasia. Growth and development were normal. An MRI performed one month earlier revealed right renal dysplasia and a funnel-shaped cystic mass located below and posterior to the bladder (Supplementary Figure S4). A tubular cystic structure (55 × 7 mm) adjacent to the mass appeared to connect with its lower portion, suggesting an ectopic ureter (Figure 3C_2_). Due to the absence of symptoms, outpatient follow-up was recommended.

**Figure 4:**
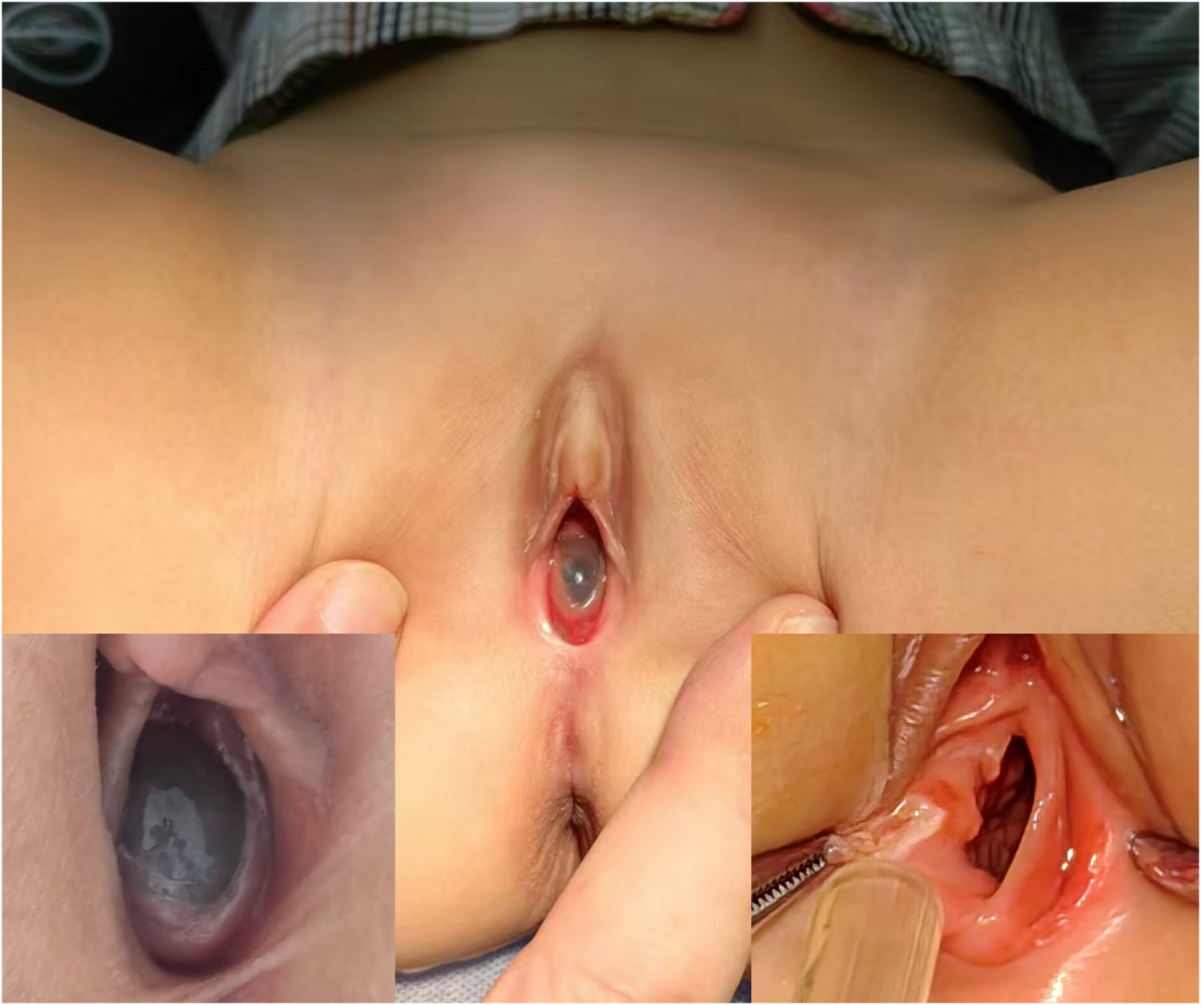
A 2-year-7-month-old girl with Gartner duct cyst involving an ectopic ureter and renal dysplasia, showing a cystic mass (approximately 2 cm in diameter) protruding between the labia minora, originating near the vaginal introitus.

On admission, a cystic mass approximately 2 cm in diameter was observed protruding from the vaginal introitus between the labia minora (Figure 4). The rest of the physical examination was unremarkable. Ultrasound revealed a cystic mass (67 × 40 × 39 mm) posterior to the bladder (Figure 2B_2_), likely originating from the vagina, and a right-sided cystic structure (28 × 14 × 24 mm) that appeared to communicate with it. A contrast-enhanced abdominal CT scan revealed an ectopic, dysplastic right kidney above the iliac vessels, cystic dilation of the distal ureter (Supplementary Figure S5A), and possible vaginal dilatation, suggesting hydrocolpos (Figure 1A_2_; Supplementary Figure S5B).

A multidisciplinary team also evaluated the case for OHVIRA syndrome with imperforate hymen. Consent was obtained for cystoscopy, vaginoscopy, and laparoscopic exploration. Under anesthesia, examination revealed a cystic mass near the vaginal opening, with normal urethral and vaginal orifices. Cystoscopy confirmed the absence of the right ureteral orifice, while the left was normal. Vaginoscopy revealed a normal vaginal wall and cervix. Incision of the cyst released clear fluid, revealing an ectopic ureteral opening. Laparoscopy was used to remove the ectopic dysplastic kidney and distal ureter. The patient recovered well and was discharged on day three. The patient's parents were satisfied with the outcome, noting the successful recovery and effective care provided. A two-month follow-up ultrasound showed no cystic masses, and the patient remained asymptomatic.

## Discussion

GDC with an ectopic ureter results from the failure of the terminal ureteric bud to separate from the mesonephric duct, preventing proper fusion with the primitive bladder and leading to ectopic insertion into the GDC (5). In OHVIRA syndrome, abnormal development of the mesonephric duct disrupts Müllerian duct fusion, causing the ureter to open into the ipsilateral obstructed hemivagina (1, 3). This embryologic overlap contributes to the imaging and clinical similarities between OHVIRA and GDC, which, especially when accompanied by renal dysplasia and ectopic ureter, pose significant challenges for preoperative diagnosis. Therefore, these overlapping imaging features emphasize the need for comprehensive imaging and a multidisciplinary approach for accurate differentiation in prepubertal patients.

Both cases were asymptomatic until treatment, whereas adolescents or adults may experience cyclic abdominal pain, urinary symptoms (e.g., infections or incontinence), or uterine bleeding (6, 7). Detailed imaging, including CT, MRI, and ultrasound, played a critical role in identifying key features of each condition. However, even with advanced imaging, intraoperative exploration was necessary to accurately diagnose the cysts and determine the proper course of treatment. In Case 1, imaging and ultrasound failed to identify uterus didelphys, leading to an uncertainty in the preoperative diagnosis. Laparoscopy and vaginoscopy ultimately confirmed the diagnosis of OHVIRA syndrome.

Case 2 had a similar clinical history and imaging findings to Case 1, initially suggesting OHVIRA syndrome. Intraoperative exploration revealed that the cyst originated from the paravaginal area, further complicating the diagnosis. Possible diagnoses included a blind-ended ectopic ureterocele, obstructed hemivaginal protrusion, Gartner duct cyst, or other mesonephric (Wolffian) duct derivatives (5, 8). However, further evaluation was needed to confirm the exact diagnosis.

Vaginoscopy identified only one cervix, and laparoscopy confirmed a single uterine body, ruling out OHVIRA syndrome. Ectopic ureteroceles are typically located distal to the trigone, inserting into the bladder neck, urethra, or other pelvic areas, often presenting with urinary incontinence depending on the function of the ipsilateral kidney (9). However, the blind-ended cyst in this case did not align with these features. Cystoscopy showed no right hemitrigone, ureteral orifice, intravesical ureterocele, or ectopic ureter opening into the urethra. Exploration of the cyst cavity revealed a wide ureteral opening at the proximal end without compression signs, ruling out this diagnosis as well. GDCs can extend alongside anterolateral vaginal wall from the proximal bladder to the perineal vestibule—consistent with this case's presentation. Unfortunately, histopathological analysis was not performed, which could have helped differentiate the cyst's origin, specifically whether it was a mesonephric or Müllerian derivative. Mesonephric (Wolffian) cysts typically consist of cuboidal or low columnar cells, whereas Müllerian derivatives are characterized by stratified squamous epithelium (2).

However, the appropriate timing of surgical intervention in OHVIRA and GDC cases remains controversial (10). OHVIRA in prepubertal patients is often discovered incidentally, whereas adolescents may present with cyclic pain, hematocolpos, or infections following menstruation (1). Treatment is typically recommended at puberty to alleviate symptoms and prevent complications such as endometriosis or infertility (11). Jang Hee Han et al. (3) reported 43 prepubertal OHVIRA cases, with six symptomatic patients undergoing vaginal septum resection and three undergoing nephrectomy for ectopic multicystic dysplastic kidney. The remaining cases did not routinely undergo septum resection due to insufficient evidence. Carmen Capito et al. (12) retrospectively studied eight prepubertal OHVIRA cases, with seven undergoing septum and dysplastic kidney resection, though symptom status was not specified. In our case, we performed transvaginal septal resection and nephrectomy safely.

For GDC, conservative management is recommended for asymptomatic cases, while excision or marsupialization is indicated for symptomatic cases with compression or infections. Sida Niu et al. (2) used fluorescein dye for complete GDC resection, while Sandra L. Emmons et al. (13) advised against resection if intact removal was not feasible, suggesting marsupialization or sclerotherapy instead. In our case, considering the patient's young age and the potential local trauma associated with complete GDC excision, a partial cyst wall resection was performed. Despite imaging suggesting renal agenesis, an ectopic ureter opening into the GDC was identified, prompting excision of the dysplastic kidney.

This study did not confirm whether the fluid in the GDC or OHVIRA cavities was urine, nor were contrast studies performed to delineate the ectopic ureteral course or identify its precise openings. Histopathological examination was also not conducted to distinguish the cyst origin (mesonephric vs. Müllerian). Moreover, the small uterine size typical of prepubertal girls limited the visualization of uterine duplication on imaging. As a result, intraoperative findings played a pivotal role in establishing the final diagnosis. Future studies incorporating histopathological confirmation, imaging enhancements, and extended follow-up are warranted to strengthen diagnostic accuracy and evaluate long-term outcomes.

## Conclusion

The overlapping embryologic origins and imaging features of OHVIRA syndrome with ectopic ureter and GDC with ectopic ureter and renal dysplasia pose significant diagnostic challenges. These cases highlight the need for careful clinical evaluation, detailed imaging, and a multidisciplinary approach for accurate differentiation and appropriate intervention. For asymptomatic cases, the benefits of prepubertal surgical treatment require long-term follow-up to assess outcomes and potential complication.

## Data Availability

The original contributions presented in the study are included in the article/Supplementary Material, further inquiries can be directed to the corresponding author.

## Supplementary material

Supplementary Figure S1 CT scan of an 8-year-old girl with OHVIRA syndrome involving a right-sided ectopic ureter, showing right renal dysplasia, compensatory hypertrophy of the left kidney (green arrow), a cystic mass (red arrow), and dilation of the ureter (blue arrow) posterior to the bladder (yellow arrow).

Supplementary Figure S2 B-ultrasound showed a right ectopic ureter in an 8-year-old girl with OHVIRA syndrome, with a cystic mass posterior to the bladder (yellow arrow) and a dilated distal right ureter (9 mm) (blue arrow) potentially communicating with the vagina.

Supplementary Figure S3 MRI showed a right ectopic ureter in an 8-year-old girl with OHVIRA syndrome, with a cystic lesion (red arrow) posterior to the bladder (yellow arrow), indicating vaginal distension and fluid accumulation, possibly due to an oblique septum (green arrow).

Supplementary Figure S4 Three-dimensional MR urography of a 2-year-7-month-old girl with Gartner duct cyst involving an ectopic ureter and renal dysplasia, showing a funnel-shaped cystic mass (red arrow) posterior to the bladder (yellow arrow), with a possible ectopic ureter (blue arrow) communicating with the lower portion of this mass.

Supplementary Figure 5 CT scan of a 2-year-7-month-old girl with a Gartner duct cyst, ectopic ureter, and renal dysplasia: showing an ectopic dysplastic right kidney above the iliac vessels (purple arrow), cystic dilation of the distal ureter (blue arrow), and possible vaginal dilatation suggesting hydrocolpos (red arrow).
