# Supplementary figures and images for: Case Report: Diagnostic overlap of OHVIRA syndrome and Gartner duct cyst: challenges in imaging and management
Source: Front Pediatr. 2025 May 22;13:1536314. doi: 10.3389/fped.2025.1536314 (PMC12139208; doi:10.3389/fped.2025.1536314)

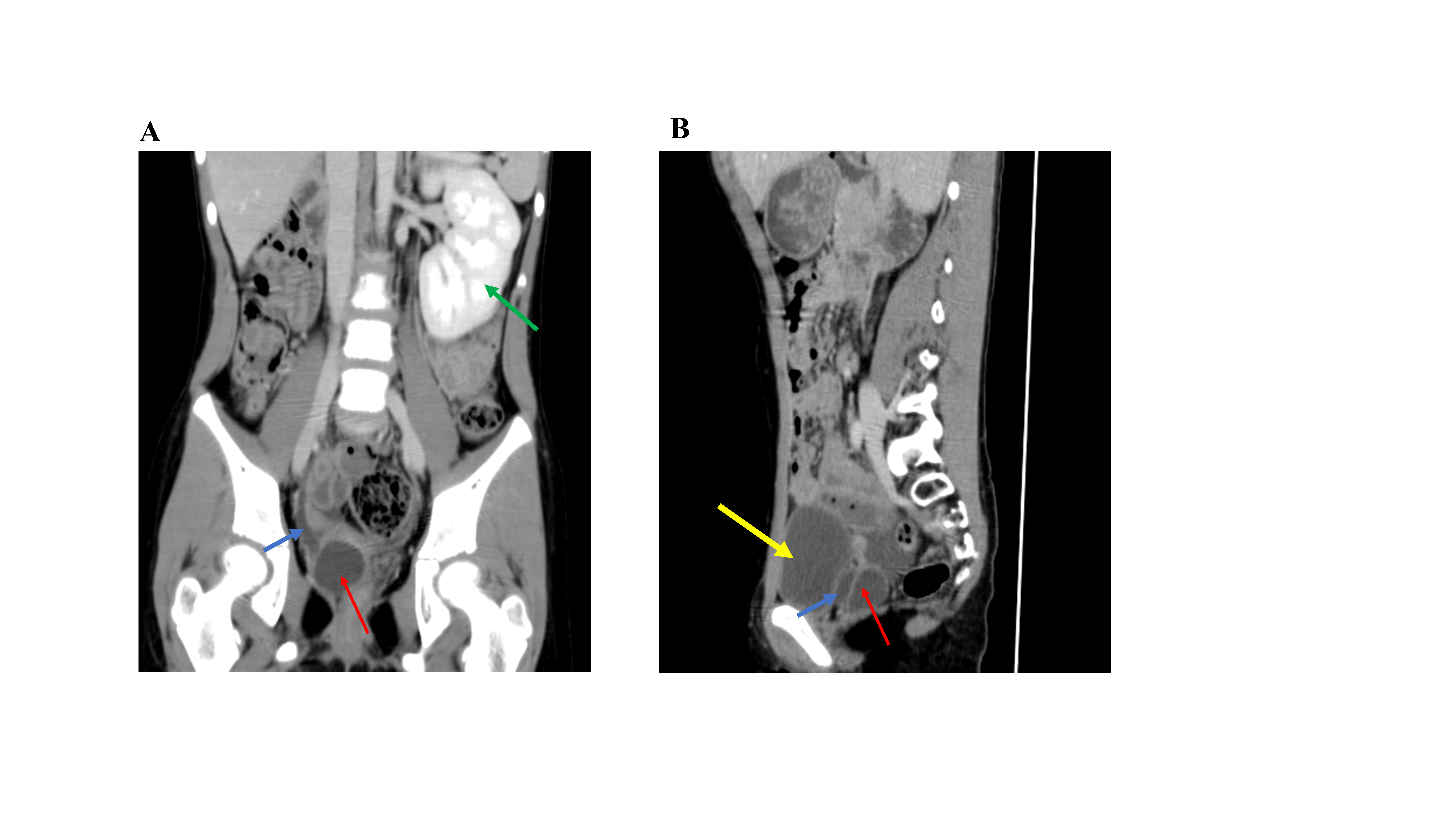

Supplement: Supplementary Figure S1 — CT scan of an 8-year-old girl with OHVIRA syndrome involving a right-sided ectopic ureter, showing right renal dysplasia, compensatory hypertrophy of the left kidney (green arrow), a cystic mass (red arrow), and dilation of the ureter (blue arrow) posterior to the bladder (yellow arrow). [file Image1.tif]

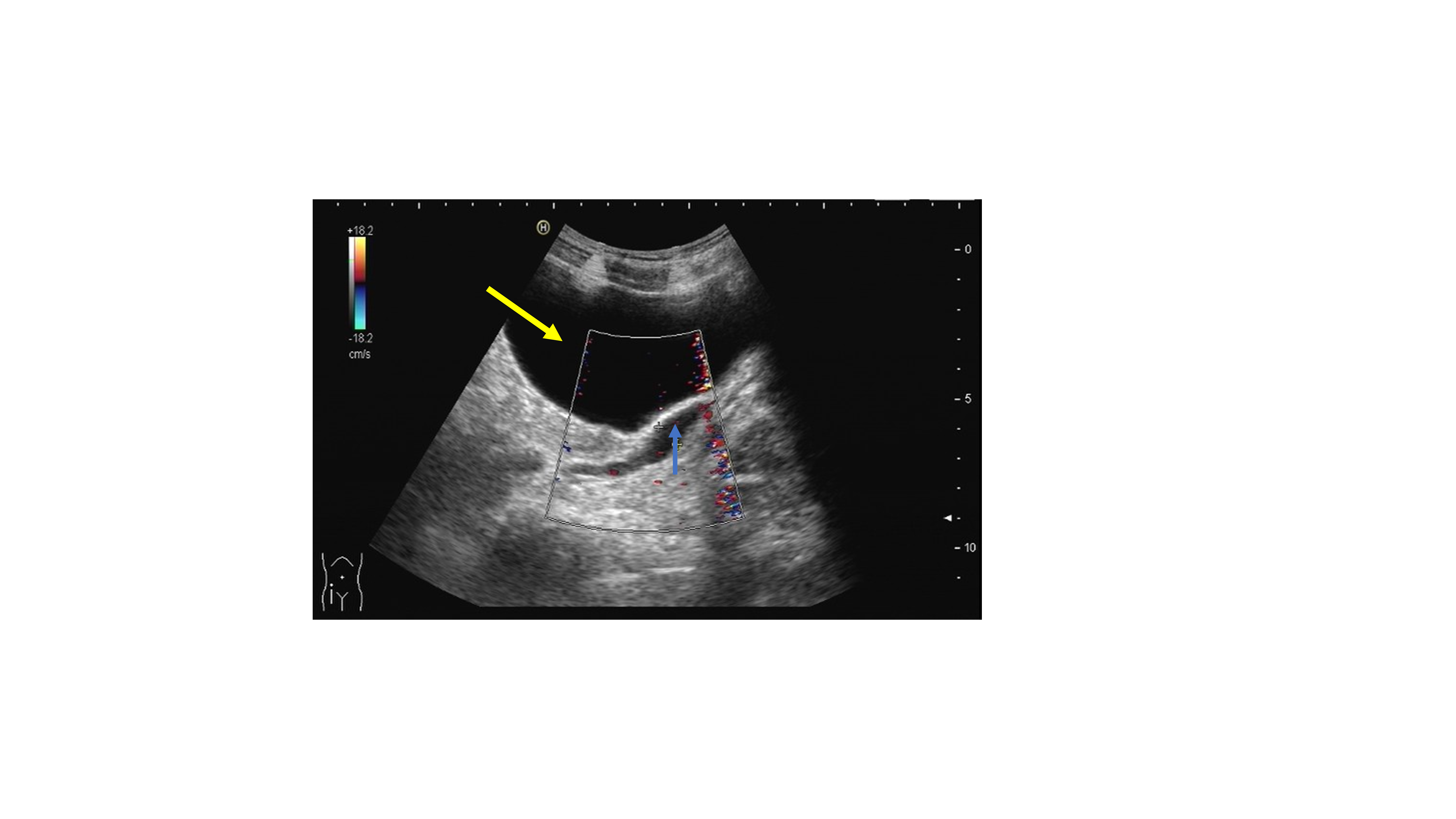

Supplement: Supplementary Figure S2 — B-ultrasound showed a right ectopic ureter in an 8-year-old girl with OHVIRA syndrome, with a cystic mass posterior to the bladder (yellow arrow) and a dilated distal right ureter (9 mm) (blue arrow) potentially communicating with the vagina. [file Image2.tif]

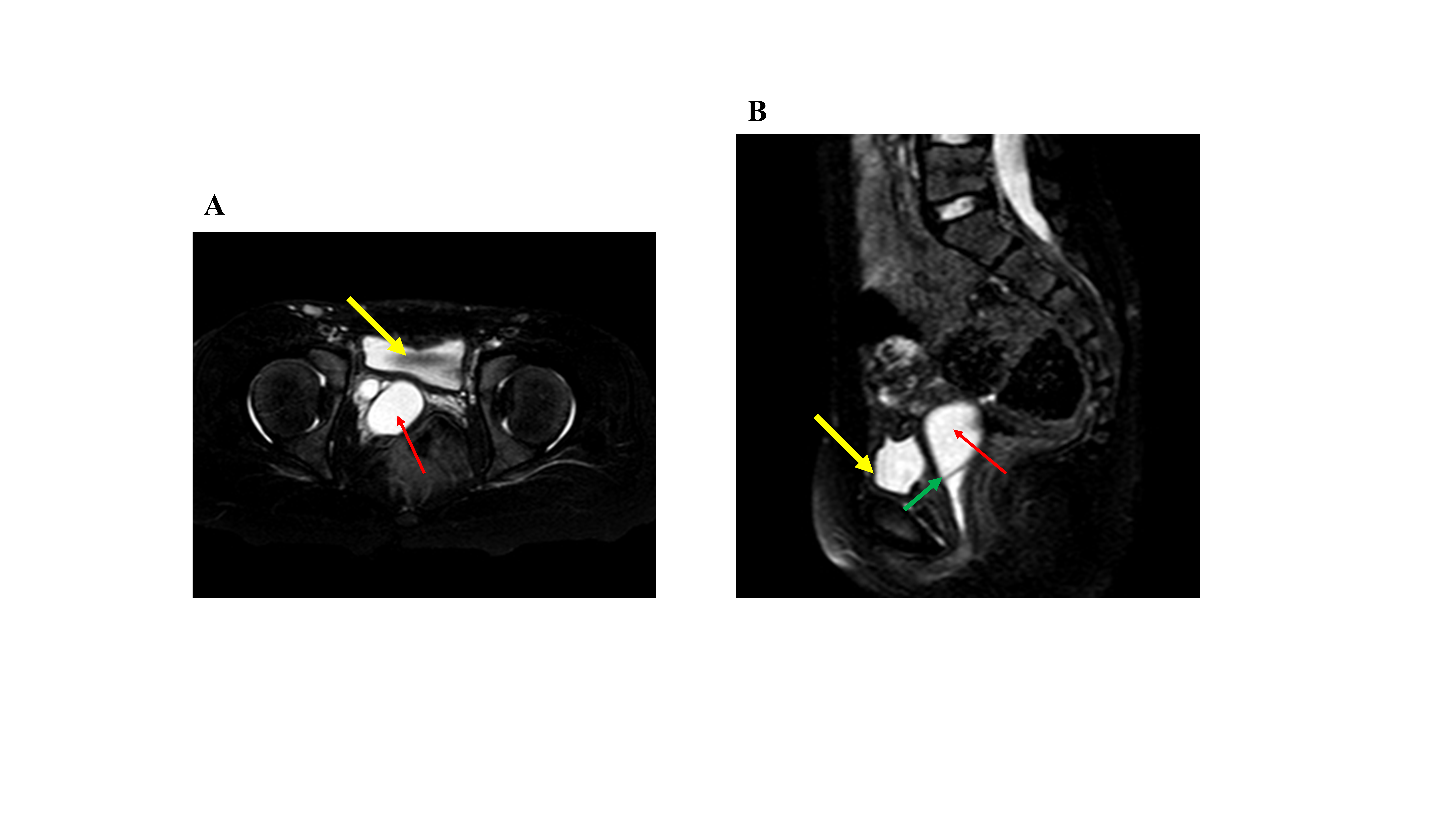

Supplement: Supplementary Figure S3 [file Image3.tif]

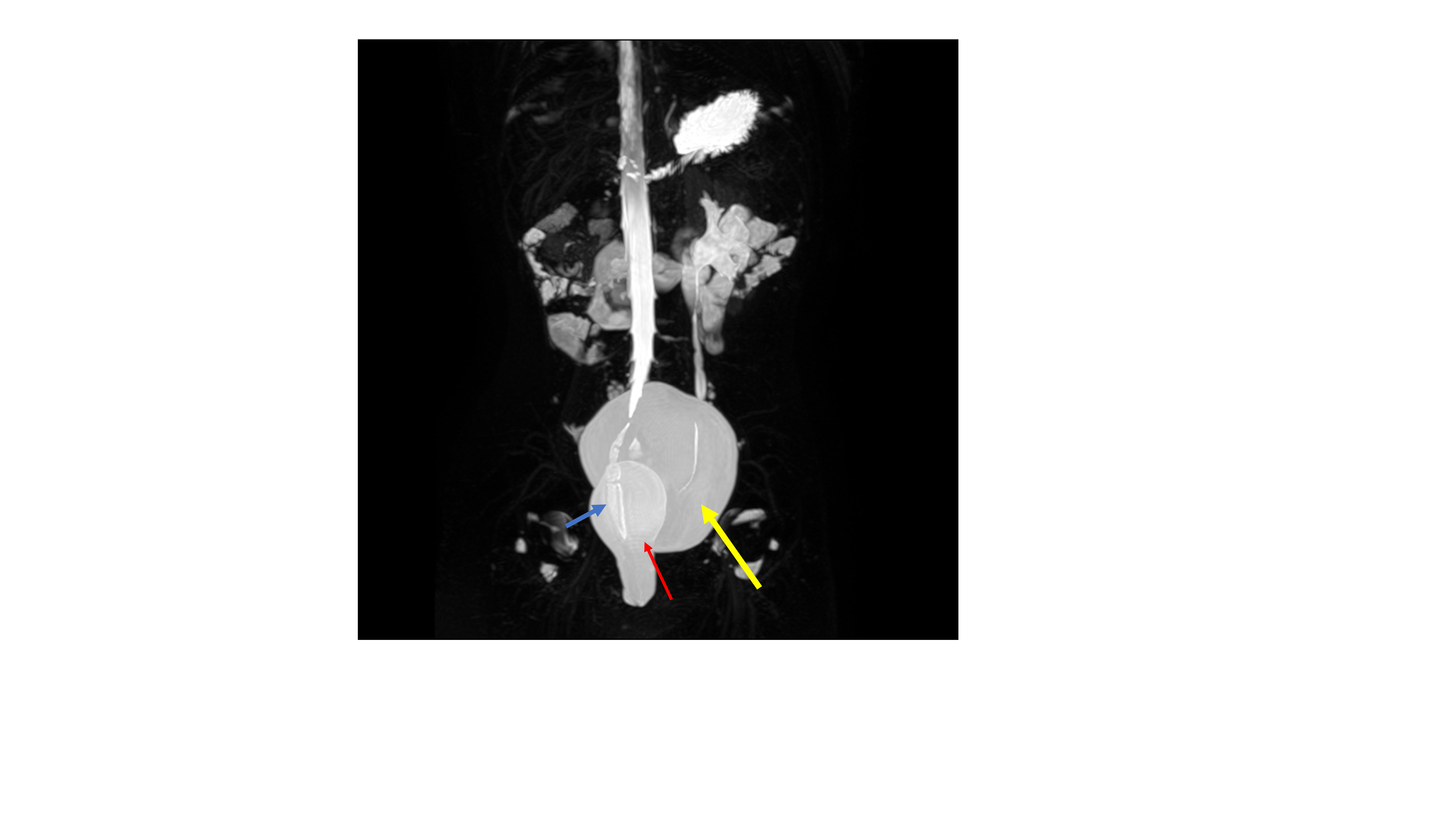

Supplement: Supplementary Figure S4 [file Image4.tif]

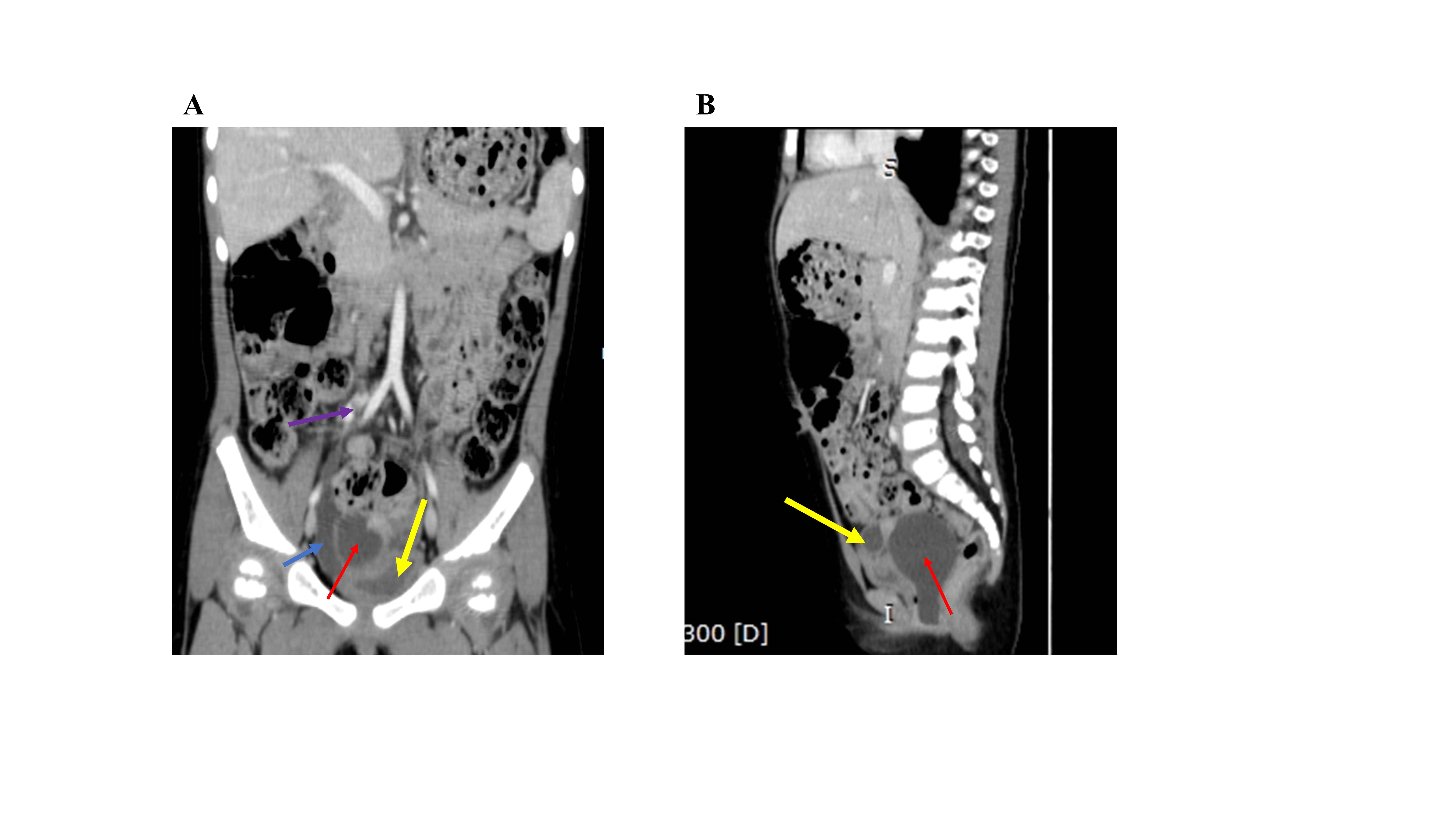

Supplement: Supplementary Figure 5 [file Image5.tif]
