# Supplementary material for: Case Report: Diagnostic overlap of OHVIRA syndrome and Gartner duct cyst: challenges in imaging and management
Source: Front Pediatr. 2025 May 22;13:1536314. doi: 10.3389/fped.2025.1536314 (PMC12139208; doi:10.3389/fped.2025.1536314)
Supplement: Supplementary file 6 [file Table1.pdf]

Supplementary Table 1. Comparison of clinical and imaging characteristics between two cases

Feature	Case 1: OHVIRA syndrome with ectopic ureter	Case 2: GDC with ectopic ureter and renal dysplasia
**Age and Gender**	8-year-old girl	2-year-7-month-old girl
**Diagnosis**	OHVIRA syndrome with right ectopic ureter	GDC with right ectopic ureter and renal dysplasia
**Clinical Presentation**	No UTI or incontinence	No UTI or incontinence
**Main Imaging Findings (CT/MRI)**	Right renal agenesis, cyst posterior to bladder, ectopic ureter opening into cyst, oblique septum	Right renal dysplasia, cyst posterior to bladder, ectopic ureter opening into cyst
**Ultrasound**	33×24 mm cyst posterior to bladder near vaginal wall	67×40 mm cyst posterior to bladder near vaginal wall
**Treatment**	Resection of vaginal septum, ureter and dysplastic kidney, favorable recovery	Resection of ureter and dysplastic kidney, cyst fenestration, favorable recovery
**Follow-up**	No vaginal fluid accumulation at 3 months	No cyst formation at 2 months

OHVIRA, Obstructed hemivagina and ipsilateral renal anomaly; GDC, Gartner duct cyst; UTI, urinary tract infection.
